# Unmet needs for hypertension diagnosis among older adults in Myanmar: secondary analysis of a multistage sampling study

**DOI:** 10.1186/s12961-022-00918-y

**Published:** 2022-11-29

**Authors:** Ikuma Nozaki, Yugo Shobugawa, Yuri Sasaki, Daisuke Takagi, Yuiko Nagamine, Poe Ei Zin, Thae Zarchi Bo, Than Win Nyunt, Min Zaw Oo, Kay Thi Lwin, Hla Hla Win

**Affiliations:** 1 Bureau of International Health Cooperation, National Center for Global Health and Medicine, 1-21-1 Toyama, Shinjuku-Ku, Tokyo, Japan; 2 Graduate School of Medical and Dental Sciences, Niigata University, Niigata, Japan; 3 Department of International Health and Collaboration, National Institute of Public Health, Saitama, Japan; 4 Graduate School of Medicine, The University of Tokyo, Tokyo, Japan; 5 Department of Family Medicine, Tokyo Medical and Dental University, Tokyo, Japan; 6 Department of Preventive and Social Medicine, University of Medicine 1, Yangon, Myanmar; 7 Department of Geriatric Medicine, Yangon General Hospital, Yangon, Myanmar; 8 University of Public Health, Yangon, Myanmar

**Keywords:** Older adults, Healthcare access, Self-rated health status, Japan Gerontological Evaluation Study (JAGES)

## Abstract

**Background:**

Hypertension is a major cause of morbidity among older adults. We investigated older adults’ access to health services in Myanmar by focusing on unmet needs in diagnosing hypertension. This study aims to identify factors associated with the unmet needs for hypertension diagnosis in the study areas of Myanmar.

**Methods:**

This is a secondary data analysis of the survey which is a cross-sectional study conducted with older adults (aged ≥ 60 years) in the Yangon and Bago regions of Myanmar. Objective indicators of health were collected, including blood pressure, height and weight. The diagnosis of hypertension was considered an unmet need when a participant’s blood pressure measurement met the diagnostic criteria for hypertension but the disease had not yet been diagnosed. Bivariate and multivariate analyses using logistic regression were performed to identify factors associated with the unmet need for hypertension diagnosis. Factors related to lifestyle habits and medical-seeking behaviour were selected and put into the multivariate model.

**Results:**

Data from 1200 people, 600 from each of the two regions, were analysed. Altogether 483 (40.3%) participants were male, 530 (44.2%) were aged ≥ 70 years, and 857 were diagnosed with hypertension based on their measured blood pressure or diagnostic history, or both, which is a 71.4% prevalence of hypertension. Moreover, 240 (20.0%) participants had never been diagnosed with hypertension. In the multivariate analysis, these unmet needs for hypertension diagnosis were significantly associated with male sex (odds ratio [OR] 1.46, 95% confidence interval [CI] 1.05–2.05), residence in the Bago region (OR 1.64, 95% CI 1.09–2.45) and better self-rated health (OR 1.70, 95% CI 1.24–2.33), but not with education, category on the wealth index or living arrangement.

**Conclusions:**

There are barriers to accessing health services for hypertension diagnosis, as evidenced by the regional disparities found in this study, and charitable clinics may decrease the financial barrier to this diagnosis.

## Background

Population ageing in Myanmar has progressed rapidly in recent years as a result of declining fertility and improving survival, similar to changes in other countries in South-East Asia [1]. In 2019, 6% of the population in Myanmar was estimated to be aged ≥ 65 years, which is less than the 7% criterion that determines an ageing society; however, this percentage is estimated to reach 13% in 2050, which approaches the criterion for an aged society (14%) [2]. Older people tend to have chronic conditions, particularly noncommunicable diseases (NCDs) such as hypertension [3]. A community-based study of older people in urban and rural areas of Myanmar found that 58% of participants had at least one chronic condition in the preceding year and that 33% reported two or more conditions, including hypertension (67%), arthritis (25%), arrhythmia (15%), coronary heart disease (14%) and diabetes (14%) [4]. In Myanmar, due to urbanization, an increasingly westernized lifestyle and economic development, the prevalence of behavioural and metabolic risk factors for NCDs—such as daily smoking, alcohol consumption, unhealthy eating habits, physical inactivity and obesity—have increased, similar to those in other Asian countries that are undergoing rapid economic development [5, 6]. In fact, the number of hospitalizations due to major NCDs in Myanmar increased 2.2-fold in the 6 years from 2012 to 2017, inclusive [7], and in 2014 the proportional mortality rate due to NCDs—such as cardiovascular diseases, cancers, chronic respiratory diseases and diabetes—among all deaths in Myanmar was 59% [8]. In Myanmar, hypertension is one of the commonest NCDs, and it is the leading risk factor for other NCDs, such as cardiovascular diseases and cerebrovascular disease. A nationwide study of 7429 people reported an estimated prevalence of hypertension among individuals aged 15–64 years of 30% and among those aged 55–64 years of 52% [9].

Early diagnosis, lifelong supportive pharmacotherapy and early detection of and effective care for complications are the mainstays of management for averting premature mortality due to the major NCDs [4]. However, the utilization of health services by older adults in Myanmar has not yet been adequately described. This study aimed to examine the unmet needs for hypertension diagnosis and the factors associated with it in urban and rural areas of Myanmar.

## Methods

### Study design and subject

Our study is a secondary analysis of data from the 2018 survey of healthy and active ageing in Myanmar. Based on the collected data from the 2018 survey, cross-sectional analysis was done. The 2018 survey was conducted using a questionnaire from the Japan Gerontological Evaluation Study (known as JAGES). The survey was a population-based multistage cluster-sample questionnaire survey conducted among community-dwelling older adults in the Yangon and Bago regions from September to December 2018 [10]. In the first stage of the survey, six townships in each region were selected using population-proportionate sampling. Townships constitute third-level administrative divisions in Myanmar, each of which has approximately 100 000 inhabitants. Townships comprise wards in urban areas and village tracts in rural areas. Consequently, 10 wards and 10 village tracts from selected townships in Yangon and Bago were chosen by population-proportionate random sampling. Finally, 10 eligible individuals aged ≥ 60 years were randomly selected from the list of older residents available in the ward or village-tract office, and they were invited to participate in the survey. Individuals were excluded if they were bedridden or had severe dementia, defined as an Abbreviated Mental Test score of ≤ 6 [11, 12]. The study recruitment process continued until the prespecified number of eligible participants in the study protocol was reached.

### Data collection

For collecting data in 2018 survey, after obtaining written informed consent, participants were interviewed in person by trained interviewers using a paper-based semi-structured questionnaire. The questionnaire was adapted from the JAGES questionnaire [13]—which encompasses health, psychological and functional factors, and social determinants of health—to the context in Myanmar; the survey was translated into the local language. Objective measurements were made during the home visit and included the participant’s body weight, height, body composition, abdominal circumference, blood pressure and grip strength. Before the survey began, interviewers were trained to use an automated sphygmomanometer (BC-757, TANITA Corporation, Tokyo, Japan) and measured blood pressure with it during the home visit. All data were double-entered in an Excel database, and discrepancies were checked against the raw data.

### Data analysis

Data were analysed with Stata/MP version 16 (Stata Corporation, College Station, TX, USA). The primary analysis calculated the unmet need for hypertension diagnosis, which was defined as occurring when the measured blood pressure was above the diagnostic criterion for hypertension, but the individual had not yet been diagnosed. Hypertension was diagnosed when for the average of two measurements systolic pressure was ≥ 140 mmHg or diastolic pressure was ≥ 90 mmHg, or both. To describe the consequences of a diagnosis of hypertension, we calculated the number of participants who had access to any kind of treatment and the number of participants who could achieve blood pressure control. The wealth index was used as an economic indicator, and participants were categorized into one of three groups—rich (20%), middle class (40%) or poor (40%)—based on an assessment of household assets using a previously reported method [14]. To identify the factors associated with the unmet need for hypertension diagnosis, bivariate analysis using a logistic regression model was conducted by including the following variables: sex, age group, residential area, educational level, marital status, category on the wealth index, whether the participant was living with a relative, and smoking, eating and walking habits. Sex, region, category in the wealth index, smoking, walking and self-rated health status were found to be significant in the bivariate analysis were included in the multivariate analysis (Model 1). We inferred that living with a relative might affect the participant’s access to health services and, therefore, this item was also included in the analysis as living with a spouse (Model 2), living with a daughter (Model 3) and living with a son (Model 4), even if it was not found to be significant in the bivariate analysis. All analyses also considered survey weighting to avoid sampling bias and to better describe the representativeness of the older population in each region. Survey weight was calculated to divide by population at township level. All analyses were conducted with the significance level set at 0.05.

### Ethics approval

This research project was approved by the Ethics Review Committee of the Department of Medical Research, Myanmar Ministry of Health and Sports (approval no. Ethics/DMR/2018/038); the Research Ethics Review Committee of the World Health Organization (protocol no. ERC.0003072); and the Ethics Board of Niigata University (Approval No. 2018-0096).

## Results

### Characteristics of participants in the survey

In total, 1200 older adults participated in this study, including 600 from the Yangon region (222 men and 378 women) and 600 from the Bago region (261 men and 339 women). Participation rate was 98.4% in Yangon and 86.5% in Bago. The characteristics of the participants are shown in Table 1. In this study, 670 (55.8%) participants were aged 60–69 years and 530 (44.2%) were aged ≥ 70 years. Age range of the study participants was between 60 and 95 years old. Participants included 387 (32.3%) with middle school or a higher level of education, 558 (46.5%) who were currently unmarried, and 480 (40.1%) and 481 (40.1%) who were categorized as poor and middle class based on the wealth index. Altogether 68 (5.7%) participants were living alone, 664 (55.3%) were living with a spouse, 535 (44.6%) were living with a son, and 707 (58.9%) were living with a daughter. With regard to current lifestyle habits related to health intentions, 288 (24.0%) were smokers, 446 (37.2%) chewed betel, 815 (67.9%) ate vegetables or fruits daily, and 361 (30.1%) walked for ≥ 1 hour every day.

**Table 1:** Characteristics of participants in study of healthy ageing, Myanmar, 2018

Characteristics	Region		Total (*N* = 1200)
	Yangon	Bago	
Sex			
Male	222	261	483
Female	378	339	717
Age group, years			
60–69	351	319	670
≥ 70	249	281	530
Education			
Below primary level	290	523	813
Middle school or above	310	77	387
Marital status			
Married	315	327	642
Unmarried currently	285	273	558
Wealth index			
Poor	64	416	480
Middle class	305	176	481
Rich	230	7	237
Living with spouse			
Yes	299	365	664
No	301	235	536
Living with son			
Yes	278	257	535
No	322	343	665
Living with daughter			
Yes	382	325	707
No	218	275	493
Living alone			
Yes	23	45	68
No	577	555	1,132
Alcohol drinking			
Teetotaller	506	446	952
Ever drank	94	154	248
Smoking			
Nonsmoker currently	502	410	912
Smoker	98	190	288
Betel chewing			
No	434	320	754
Yes	166	280	446
Consumes fruits and vegetables			
Daily	397	418	815
Less than daily	203	182	385
Daily walking			
≥ 1 hour	163	198	361
< 1 hour	437	402	839
Body mass index (kg/m^2^)			
Underweight (< 18.5)	85	237	322
Normal (18.5–25)	275	273	548
Overweight (≥ 25)	240	90	330
Social participation			
No	497	544	1,041
Yes	103	56	159
Social cohesion			
Less	213	155	368
Much	387	445	832
Social support			
Less	204	192	396
Much	396	408	804
Self-rated health			
Fair or poor	367	479	846
Excellent or good	233	121	354
Blood pressure			
Normal range	294	257	551
Hypertension^a^	306	343	649
Ever diagnosed as hypertensive			
Yes	334	283	617
No	258	295	553
Unsure	8	22	30

^a^Hypertension was diagnosed if systolic blood pressure was ≥ 140 mmHg and/or diastolic blood pressure was ≥ 90 mmHg

### Unmet need for hypertension diagnosis

There were 551 (45.9%) normotensive participants and 649 (54.1%) hypertensive participants (defined as an average of two blood pressure measurements of ≥ 140/90 mmHg), of whom 553 (46.1%) had never been diagnosed with hypertension in a medical setting. The measured blood pressure values of 649 (54.1%) participants met the diagnostic criteria for hypertension. Adding the 208 participants with normal blood pressure measurements but a history of being diagnosed with hypertension, 857 participants were categorized as hypertensive based on their measured blood pressure or diagnostic history, a crude prevalence of 71.4% (857/1200). The estimated prevalence calculated by weighted value was 71.6% (95% confidence interval [CI]: 68.7% to 74.4%). Among those 857 participants, 617 (crude prevalence: 72.0%) had been diagnosed with hypertension and 571 (crude prevalence: 66.6%) had access to some kind of treatment, including traditional medicine. The corresponding estimated values analysed by weighted sample were 73.7% (95% CI: 70.5% to 76.7%) and 68.4% (95% CI: 64.5% to 72.2%), respectively. However, the measured blood pressure of only 186 participants (crude prevalence: 32.6%; prevalence estimated by weighted sample: 32.8%; 95% CI: 28.0% to 37.6%) was controlled in the normotensive range (data not shown).

### Factors associated with the unmet need

In our primary analysis of the unmet need for hypertension diagnosis, the measured blood pressure of 240/1200 participants (crude prevalence: 20.0%; estimated prevalence by weighted sample: 18.8%; 95% CI: 16.6% to 21.1%) was above the diagnostic criterion for hypertension, although they had never been diagnosed. The bivariate analysis of the factors associated with the unmet need for hypertension diagnosis showed that the following characteristics were significant (Table 2): male sex (odds ratio [OR]: 1.69; 95% CI: 1.24 to 2.31), residence in the Bago region (OR: 1.77; 95% CI: 1.31 to 2.39), being categorized as poor or rich compared to middle class on the wealth index (OR: 1.23; 95% CI: 0.92 to 1.64 and OR: 0.62; 95% CI: 0.40 to 0.96), being a smoker (OR: 1.54; 95% CI: 1.15 to 2.06), walking for ≥ 1 hour every day (OR: 1.48; 95% CI: 1.10 to 1.99) and having excellent or good self-rated health (OR: 1.62; 95% CI: 1.24 to 2.12). The multivariate analysis was adjusted for sex, region, category in the wealth index, smoking, walking and self-rated health status, as these variables were found to be significant in the bivariate analysis. The results showed that male sex (OR: 1.46; 95% CI: 1.04 to 2.03), residence in the Bago region (OR: 1.76; 95% CI: 1.15 to 2.69) and having excellent or good self-rated health (OR: 1.70; 95% CI: 1.24 to 2.33) remained significant (Table 3, Model 1). The results of the analyses that included living with a relative are shown in Models 2, 3 and 4 in Table 3. On multivariate analysis, we found that living with a relative was not a significant factor affecting the unmet need for hypertension diagnosis.

**Table 2:** Bivariate analysis of the unmet need for hypertension diagnosis, Myanmar

Characteristics	Unmet need for hypertension diagnosis		Odds ratio (95% CI)
	Undiagnosed hypertension	Normotensive or diagnosed hypertension	
Sex			
Male	122	361	1.69 (1.24 to 2.31)
Female	118	599	Ref.
Age group, years			
60–69	127	543	0.84 (0.62 to 1.13)
≥ 70	113	417	Ref.
Residence			
Yangon	94	506	Ref.
Bago	146	454	1.77 (1.31 to 2.39)
Education			
Below primary level	173	640	1.22 (0.89 to 1.69)
Middle school or above	67	320	Ref.
Marital status			
Married	135	507	1.12 (0.83 to 1.51)
Unmarried currently	105	453	Ref
Wealth index			
Poor	110	370	1.23 (0.92 to 1.64)
Middle class	98	383	Ref.
Rich	32	205	0.62 (0.40 to 0.96)
Living with spouse			
Yes	140	524	1.14 (0.83 to 1.55)
No	100	436	Ref.
Living with son			
Yes	99	436	0.79 (0.57 to 1.09)
No	141	524	Ref.
Living with daughter			
Yes	140	567	0.93 (0.70 to 1.24)
No	100	393	Ref
Living alone			
Yes	12	56	1.05 (0.55 to 2.01)
No	228	904	Ref.
Alcohol drinking			
Teetotaller	179	773	0.70 (0.48 to 1.00)
Ever drank	61	187	Ref.
Smoking			
Nonsmoker currently	165	747	Ref
Smoker	75	213	1.54 (1.15 to 2.06)
Consumes fruits and vegetables			
Daily	173	642	1.38 (0.97 to 1.95)
Less than daily	67	318	Ref.
Daily walking			
≥ 1 hour	88	273	1.48 (1.10 to 1.99)
< 1 hour	152	687	Ref.
Body mass index (kg/m^2^)			
Underweight or normal (< 25)	88	273	1.13 (0.80 to 1.59)
Overweight (≥ 25)	152	687	Ref.
Social participation			
No	212	829	1.19 (0.77 to 1.82)
Yes	28	131	Ref.
Social cohesion			
Less	71	297	090 (0.64 to 1.26)
Much	169	663	Ref.
Social support			
Less	76	320	0.94 (0.66 to 1.34)
Much	164	640	Ref.
Self-rated health			
Fair or poor	148	698	Ref.
Excellent or good	92	262	1.62 (1.24 to 2.12)

*CI* confidence interval, *Ref.* reference category

**Table 3:** Multivariate analysis for the unmet need for hypertension diagnosis, Myanmar

Characteristics	Adjusted odds ratio (95% CI)			
	Model 1	Model 2	Model 3	Model 4
Sex				
Male	1.46 (1.04 to 2.03)	1.54 (1.05 to 2.27)	1.46 (1.05 to 2.03)	1.46 (1.05 to 2.04)
Female	Ref.	Ref.	Ref.	Ref.
Residence				
Yangon	Ref.	Ref.	Ref.	Ref.
Bago	1.76 (1.15 to 2.69)	1.78 (1.17 to 2.73)	1.76 (1.15 to 2.70)	1.74 (1.13 to 2.66)
Wealth index				
Poor	0.93 (0.63 to 1.36)	0.93 (0.63 to 1.36)	0.93 (0.63 to 1.37)	0.92 (0.63 to 1.35)
Middle class	Ref.	Ref.	Ref.	Ref.
Rich	0.71 (0.45 to 1.13)	0.72 (0.45 to 1.14)	0.71 (0.45 to 1.12)	0.69 (0.44 to 1.10)
Smoking				
Nonsmoker currently	Ref.	Ref.	Ref.	Ref.
Smoker	1.21 (0.90 to 1.62)	1.21 (0.89 to 1.63)	1.20 (0.89 to 1.63)	1.19 (0.88 to 1.61)
Daily walking				
≥ 1 hour	1.26 (0.93 to 1.73)	1.29 (0.94 to 1.77)	1.28 (0.93 to 1.75)	1.27 (0.93 to 1.74)
< 1 hour	Ref.	Ref.	Ref.	Ref.
Self-rated health				
Fair or poor	Ref.	Ref.	Ref.	Ref.
Excellent or good	1.70 (1.24 to 2.33)	1.71 (1.25 to 2.34)	1.70 (1.24 to 2.33)	1.73 (1.26 to 2.36)
Living with spouse				
Yes		Ref.		
No		1.18 (0.81 to 1.71)		
Living with daughter				
Yes			Ref.	
No			0.96 (0.71 to 1.29)	
Living with son				
Yes				Ref.
No				1.30 (0.93 to 1.83)

*CI* confidence interval, *Ref.* reference category

## Discussion

### Prevalence of hypertension in older adults in Myanmar

This study estimated a 71% prevalence of hypertension among participants aged ≥ 60 years based on their diagnostic history (51%), blood pressure measured at the home visit (46%), or both. This prevalence is slightly higher than that of 67.3% reported for hypertension in a study that was conducted in 2016 in Bago and Mon states among adult participants aged ≥ 60 years [4]. Although that study was conducted among participants of different age groups at different study sites, other reports estimated the prevalence of hypertension to be 30.1% among adults aged 15–64 years in Myanmar in 2007 [9] and 51% among adults aged 40–99 years in the Mon state, and the Ayeyarwaddy and Bago regions in 2017 [15]. As the prevalence of hypertension often increases with age, it seems natural that the prevalence would differ among age groups in the studies. Importantly, Htet et al. reported a significant increase in the age-standardized prevalence of hypertension, from 26.7% in 2004 to 34.6% in 2014, from the results of two cross-sectional studies among 25–74-year-old adults in the Yangon region [16]. Those results suggested an increasing need for programmes to control hypertension.

### Health service coverage

Berry et. al. proposed a hypertension care cascade by subcategorizing the population with hypertension as follows: unscreened and undiagnosed, screened but undiagnosed, diagnosed but untreated, treated but uncontrolled, and treated and controlled [17]. As Tanahashi first proposed in 1978, the measurement of service coverage can be categorized in five important stages that successively lead to a desired health intervention: availability coverage (i.e. the people for whom the service is available), accessibility coverage (i.e. the people who can use the service), acceptability coverage (i.e. the people who are willing to use the service), contact coverage (i.e. the people who use the service) and effectiveness coverage (i.e. the people who receive effective care) [18]. This model has been widely used for evaluating the coverage of health services [19–22]. The combination of contact coverage and effective coverage is classified as actual coverage. The model helps us analyse the gaps that need to be bridged to ensure better service coverage.

### Unmet need for diagnosis of hypertension

In our study, among participants categorized as hypertensive, 74% had been diagnosed with hypertension, 68% had access to some kind of treatment, and 22% had blood pressure controlled in the normotensive range. These results suggest that there is a gap in diagnosis: most of those who are diagnosed can obtain treatment, but their hypertension remains uncontrolled. Moreover, Htet et al. reported that the rates of treatment and control of hypertension did not change from 2004 to 2014, despite the diagnosis of hypertension increasing from 19.4% to 27.8% during that period [16]. Therefore, we defined the unmet need for hypertension diagnosis as the proportion of participants whose blood pressure was above the diagnostic cut-off for hypertension but who had not yet been diagnosed, and we analysed the associated factors.

### Factors associated with unmet need for hypertension diagnosis

The commonly reported determinants of health-seeking behaviour in older adults are sex, age, education, socioeconomic status, residential area (urban or rural) and registration under the public system to support people’s health, including health insurance [4, 23–26]. In our study, sex, residential area and self-rated health status were associated with the unmet needs for hypertension diagnosis. Although sex is a well-known determinant of health-seeking behaviour, reports vary as to which sex is more likely to seek care. For instance, a study in Bangladesh reported more health-seeking behaviour among males, whereas a report from Brazil found more health-seeking behaviour among females [23, 26]. A study in Myanmar on multimorbidity and health-seeking behaviour among older people reported a higher prevalence of multimorbidity among older women, but it listed only residence in rural areas and out-of-pocket payments as hindering participants from consulting doctors [4]. However, the study reported that older women were more likely to be widowed, separated, divorced or unemployed than older men, suggesting that women might have less access to healthcare. Nonetheless, the results of our study showed that the unmet needs for hypertension diagnosis are more common among males and were not associated with the participants category on the wealth index.

Living in a rural area was strongly associated with an unmet need for hypertension diagnosis, similar to findings in a previous report [4]. This may reflect disparities in access to medical services between urban and rural areas [27]. Self-rated health status was another factor associated with the unmet need for a diagnosis of hypertension. The 29.5% of the study population who categorized their health status as excellent or good was similar to the 33% of the population with a similar health status in a previous study in Myanmar [28]. Furthermore, poor self-reported health is associated with multimorbidity, but not with health-seeking behaviour. Our results suggest that individuals without subjective symptoms and with self-rated health status of excellent or good did not have access to healthcare, and thus, their hypertension was not diagnosed.

Interestingly, the wealth index was not significant in multivariate analysis in our study, whereas many other studies have reported that poverty and medical expenditure are factors that hinder medical access among older adults [4, 23–26]. In our study, 91% of participants who were diagnosed with hypertension had access to some kind of treatment, although only 33% achieved blood pressure control. Latt et. al. reported that in Myanmar, many charitable hospitals are run by private-sector agents [27]. This suggests that hypertension could be diagnosed and treatment started at a charitable clinic, thus alleviating financial concerns for patients, although they might not be able to continue treatment due to limited medical resources, such as antihypertensive medicine. To decrease unmet need for hypertension diagnosis and treatment, medical access and sustainable clinic visit should be assured.

Living arrangements are a well-known determinant of health-seeking behaviour among older adults and play a key role in their use of formal and informal care [29, 30]. In Myanmar, adult children or other relatives who look after older people play a major part in caring for older adults [1, 4, 28]. However, in this study, living with a relative was not significant with regard to the unmet need for hypertension diagnosis. A comparative study of the living arrangements and psychological health of older adults in Myanmar, Viet Nam and Thailand showed that living with a child of a culturally preferred sex significantly improved the emotional health of Vietnamese and Thai elders, despite the finding of almost no significant difference in psychological well-being of older adults in Myanmar across various living arrangements [31]. This study highlights the importance of cultural nuances for theories about the nature of the relationship between living arrangements and psychological health in older adults that might extend to their health-seeking behaviour. Moreover, living arrangements in Myanmar have been changing rapidly with economic development, similar to the changes seen in Thailand, where elders are more likely not to live with their children and to receive substantial remittances from children whom they do not live with [32]. Thus, further studies should be considered to identify the influence of cultural nuances on living arrangements and the effects on the health-seeking behaviour of older adults.

## Strength and limitation of the study

A strength of our study is the large data set used in the analysis, the population-based design and the use of random sampling, as well as a high participation rate of 85%. However, this study also has several limitations. First, the generalizability of the findings beyond the Yangon and Bago regions of Myanmar is unknown—that is, they may be representative only of these two regions. Myanmar is a multiethnic country that is characterized by different cultures and customs in various regions and states. Second, this analysis adopted a cross-sectional design, and thus, causal relationships between sociodemographic factors and unmet needs for hypertension diagnosis could not be determined. The causal relationship should be investigated in a cohort study to explore potential causality. Third, all data other than the objective measurements made by trained interviewers—including weight, height and blood pressure—were collected through a questionnaire and, therefore, rely on accurate reporting by the participants, conferring a self-report bias. Despite these limitations, this study identified critical and potential factors that influence the unmet need for hypertension diagnosis in older adults in two regions in Myanmar.

## Conclusions

We found that the prevalence of hypertension among older adults in the Yangon and Bago regions was 71.6%. Among the hypertensive participants, 72% had been diagnosed previously; 93% of those who had been diagnosed had access to some kind of treatment, including traditional medicine, but only 33% of those who were treated had achieved blood pressure control in the normal range. We categorized the remaining 28% of the study population with hypertension that was undiagnosed (18.8% of participants) as representing the unmet need for hypertension diagnosis, which was significantly associated with male sex, residence in Bago and excellent or good self-rated health, but was not associated significantly with educational level, category on the wealth index and living arrangements.

In conclusion, there are barriers to accessing health services that hinder the diagnosis of hypertension, as evidenced by regional disparities, which charitable clinics may address by reducing the financial barrier to diagnosis. Individuals without subjective symptoms and with excellent or good self-rated health status might have less access to healthcare and, thus, may not be diagnosed with hypertension.

## Data Availability

The data sets generated and analysed for this study are available from the corresponding author on reasonable request.

## Abbreviations

CI: Confidence interval
JAGES: Japan Gerontological Evaluation Study
NCDs: Noncommunicable diseases
OR: Odds ratio
